# Apology and Restitution: The Psychophysiology of Forgiveness After Accountable Relational Repair Responses

**DOI:** 10.3389/fpsyg.2020.00284

**Published:** 2020-03-13

**Authors:** Charlotte V. O. Witvliet, Lindsey Root Luna, Everett L. Worthington, Jo-Ann Tsang

**Affiliations:** ^1^Psychology Department, Hope College, Holland, MI, United States; ^2^Department of Psychology, Virginia Commonwealth University, Richmond, VA, United States; ^3^Baylor University, Department of Psychology and Neuroscience, Waco, TX, United States

**Keywords:** forgiveness, accountability, apology, restitution, heart rate, rate pressure product, facial electromyography, emotion

## Abstract

Apology and restitution each represents wrongdoers’ accountable repair responses that have promoted victims’ self-reported empathy and forgiveness in crime scenario research. The current study measured emotional and stress-related dependent variables including physiological measures, to illuminate the links between predictors of forgiveness and health-relevant side effects. Specifically, we tested the independent and interactive effects of apology and restitution on forgiveness, emotion self-reports, and facial responses, as well as cardiac measures associated with stress in 32 males and 29 females. Apology and restitution each independently increased empathy, forgiveness, gratitude, and positive emotions, while reducing unforgiveness, negative emotion, and muscle activity above the brow (corrugator supercilii, CS). The presence of a thorough apology—regardless of whether restitution was present—also calmed heart rate, reduced rate pressure products indicative of cardiac stress, and decreased muscle activity under the eye (orbicularis oculi, OO). Interactions pointed to the more potent effects of restitution compared to apology for reducing unforgiveness and anger, while elevating positivity and gratitude. The findings point to distinctive impacts of apology and restitution as factors that foster forgiveness, along with emotional and embodied changes relevant to health.

## Introduction

An emerging literature provides evidence that victims are more forgiving if they receive an apology (see Fehr et al., 2010) or restitution (Carlisle et al., 2012; Witvliet et al., 2020) or both in combination (Kiefer et al., 2020). The present investigation extends this work by also examining emotional and embodied responses to apology and restitution, with implications for the growing literature on forgiveness and its physiological side effects as health pathways (Witvliet et al., in press).

We conceptualize interpersonal offenses as relational injustices for which transgressors are accountable to those harmed through their actions or failures to act (Witvliet, 2020). Whereas transgressions are violations of expectations for responsible interpersonal behavior (e.g., that a person will not cause harm), apology may signal the offender’s accountable responsibility-taking for wrongdoing against the victim, and restitution may represent tangible and quantifiable recompense for the injustice (Witvliet et al., 2020). In this way, apology and restitution may represent relational and restorative responses that reduce the gap a victim perceives between injustice experienced and the justice desired (see Witvliet et al., 2008b). Reducing this injustice gap is associated with reductions in negative and aroused affect such as fear, sadness, and anger that are part of unforgiveness (Exline et al., 2003; Worthington, 2006; Witvliet et al., 2008b; Davis et al., 2016)—while also elevating positive and prosocial responses of gratitude, empathy, and forgiveness with associated emotional and physiological change (e.g., Witvliet et al., 2010, 2019).

Prior research has found that apology and restitution can each foster forgiveness. In experiments using a burglary scenario with both students and a community sample, Witvliet et al. (2020) found that experimental manipulations of apology and restitution independently prompted greater self-reported empathy and forgiveness while decreasing unforgiving motivations such as avoidance and revenge. Carlisle et al. (2012) found self-reported and behavioral evidence that apology and restitution each prompted forgiveness-consistent responses for a lab study offense of unfair raffle ticket distribution. Specifically, receiving an apology note prompted higher self-reported forgiveness, and receiving restitution prompted a behavioral forgiveness-oriented response (i.e., higher distribution of raffle tickets). Lab-based apology research showed that including restitution-oriented information—communicating that a lab offense was fake—promoted more forgiving responses (Zechmeister et al., 2004) and lower judgments of irresponsibility (Ohbuchi et al., 1989). In addition, Jeter and Brannon (2018) found that apologies that included an expressed desire to engage in restitution were particularly effective for inducing forgiveness.

Although no known studies have assessed the affective physiology associated with receiving restitution, three studies have addressed apology and stress-related cardiovascular responses (Anderson et al., 2006; Whited et al., 2010; Kubo et al., 2012). Both Anderson et al. (2006) and Whited et al. (2010) verbally harassed participants in the lab while they completed challenging arithmetic exercises. Anderson et al. (2006) found that the absence of an apology was associated with poor recovery from systolic blood pressure elevations in highly hostile participants. Specifically, 5 min into a recovery period, participants high in hostility who received no apology showed impaired systolic blood pressure recovery, moderate recovery for a pseudo-apology lacking remorse, and best recovery for a good apology. After 10 min of recovery, hostile participants who received no apology still had higher systolic blood pressure than those who received a pseudo-apology or a good apology (Anderson et al., 2006).

In the laboratory of Whited et al. (2010), receiving an apology versus no apology improved heart rate (HR) variability, indicative of improved parasympathetic nervous system engagement and self-regulation during the recovery period. Receiving an apology was also associated with diastolic and mean arterial blood pressure recovery, but an interaction with sex revealed that this pattern occurred only in women. Men showed the opposite blood pressure responses to receiving an apology for lab-based harassment. Sex did not predict responses to apology for self-reported hostility or positive affect.

In a study by Kubo et al. (2012), Japanese students received insulting written feedback on an essay, followed by either a simple written apology for the negative feedback or no apology. Apology groups did not differ in negative or positive emotion or in their skin conductance patterns, suggesting that sympathetic nervous system engagement did not differ across groups. However, the participants in the no-apology condition showed more anger and asymmetry in frontal brain activity indicative of approach motivation. By contrast, participants in the simple apology condition did not show this asymmetry in central nervous system functioning, did not show an increase in anger, and were buffered against HR reactivity.

Increasingly, forgiveness has been conceptualized within a multidimensional emotion framework that includes verbal–cognitive, behavioral, and physiological responses (see Witvliet and McCullough, 2007; Witvliet, 2020). Forgiving and unforgiving conditions have produced differentiated physiological reactivity and recovery patterns (e.g., Witvliet et al., 2001; Lawler et al., 2003). Worthington (2006) has further described emotional forgiveness as a process in which positive other-oriented affective responses (e.g., compassion or love) supplant the negative affective responses that characterize unforgiveness (e.g., vengeful or avoidant motives, anger, and fear) and are associated with stress. Whereas emotionally forgiving a loved one would restore more positive affect, the emotional change involved in forgiving a stranger may move toward affective neutrality (Worthington, 2006; Witvliet and Root Luna, 2018).

These emotional responses are important because variations in *valence* (negative–positive hedonic tone) and *arousal* (activation level) have been identified as pivotal axes for organizing physiological reactivity patterns. Using a 2 Valence (negative, positive) × 2 Arousal (low, high) emotional imagery design that assessed physiological reactivity from pretrial relaxations baselines to emotional imagery periods, Witvliet and Vrana (1995) found main effects of valence and arousal on specific measures. Specifically, they found that negatively valent emotional imagery conditions prompted greater activation of the brow muscle (corrugator supercilii, CS), compared to positively valent emotional imagery conditions. In the same study, the more arousing emotional imagery conditions prompted greater activation of the muscle under the eye (orbicularis oculi, OO) and greater increases in HR, compared to the less arousing emotional imagery conditions. In other research, rate pressure product scores (i.e., HR and systolic blood pressure multiplied)—considered indicative of myocardial oxygen demand—have been found to become elevated when people experience stress (Kitamura et al., 1972). Stress is high arousal and negative. Using a crime scenario as a stressor, Witvliet et al. (2008b) found that in the absence of either justice or forgiveness, rate pressure product scores were significantly elevated, whereas retributive justice had a calming effect (Witvliet et al., 2008b). Forgiveness, whether measured as a trait (Lawler-Row et al., 2008) or as a state (Lawler et al., 2003), showed expected inverse relationships with rate pressure product scores. Collectively, the physiological measures have been shown to vary with emotion during imagery (Witvliet and Vrana, 1995), to be indicative of stress (Kitamura et al., 1972), and to be responsive in paradigms that assess emotion in contexts of unforgiveness, forgiveness (Witvliet et al., 2001), and justice (Witvliet et al., 2008b).

In the current experiment, we measured physiology and affective self-reports to test whether apology or restitution (or both) would have reliable effects, an approach vital to theorizing about unforgiveness and forgiveness as emotional processes. Using a psychophysiological paradigm, we tested apology and restitution in a 2 Apology (absent and present) × 2 Restitution (absent and present) design within participants. Because physiological baselines and reactivity patterns differ between people and require very large samples to test between-group manipulations, repeated-measures designs within people have been used in research on justice, forgiveness, and emotion (e.g., Witvliet et al., 2008b). The within-participant design allows each person to serve as his or her own control, yielding a stronger test of condition effects on physiology. Measuring physiology continuously shows how each condition type affects physiology, allowing us to create a difference from the pretrial baseline metric for each trial’s imagery and recovery periods. By measuring multiple trials within each condition (e.g., Apology), participants can focus exclusively on one type of condition at a time, minimizing interference across conditions. Finally, condition orders can be counterbalanced across participants using a Latin square design to control for order effects.

This experiment incorporated affective self-reports used in basic emotion research (e.g., Witvliet and Vrana, 1995) and research examining forgiveness through the lens of emotion (Witvliet et al., 2001). These included ratings of valence and arousal, as well as perceived control, which has shown increases as emotion becomes more positive in valence and lower in arousal (Witvliet and Vrana, 1995). We measured self-reported fear, sadness, and anger, consistent with theorizing about unforgiveness (Worthington, 2006). We also used single-item measures of empathic perspective-taking, forgiveness, and gratitude as prosocial responses relevant to the affective experience of receiving an apology and restitution (Witvliet et al., 2001, 2008b).

We hypothesized the following, anticipating additive effects of apology and restitution based on other research (Witvliet et al., 2020):

(A) Apology and restitution would each decrease unforgiveness and associated negative and aroused emotion, as evident in
  (1) Lower self-reported unforgiveness scale scores and ratings of negative valence, arousal, anger, fear, and sadness.
  (2) Lower CS and OO reactivity.
  (3) Lower HR and rate pressure product reactivity.
(B) Apology and restitution would increase forgiveness and positive, prosocial emotions toward the offender, as evident in
  (1) Higher empathy and Positive Responses to the Offender (PRO) scale scores.
  (2) Higher ratings of empathy, forgiveness, and gratitude.

## Materials and Methods

### Participants

Undergraduate students (32 males, 29 females) in the midwestern region of the United States completed written informed consent and participated in this Human Subjects Review Board–approved experiment as one way to satisfy a requirement to learn about research. All participants were 18 years or older (*M* = 18.9, *SD* = 0.8). Of the participants, 54 were white, 6 were Asian or Asian-American, and 1 was African-American. Data collection was completed prior to any data analysis. Regarding sample size, a previous study using repeated measures and psychophysiology in a justice-oriented paradigm found effects in 56 participants (27 males, Witvliet et al., 2008b). Given the potential for data loss in physiological studies and challenges with undergraduate sign-ups, we aimed for a sample size of 60. Data collection notes about equipment failure or movement artifacts (e.g., coughing) were cross-checked with visual inspection of the data. In actuality, minimal missing data occurred: one missing for anger, sadness, and gratitude ratings, and OO electromyogram (EMG); two missing fear ratings and CS EMG; three missing valence and arousal ratings; and six missing blood pressure data due to equipment failure. In conducting a *post hoc* power analysis, we determined that with α = 0.05 and a power of 0.80, we would be able to detect a repeated-measures effect size as small as an SPSS-generated $\eta_{\text{p}}^{2}$ = 0.06 (*f* = 0.25) given our total collected sample size (Faul et al., 2009). Thus, we report how we determined our sample size, all data exclusions, all manipulations, and all measures in the study.

### Stimulus Materials

**FIGURE 1:**
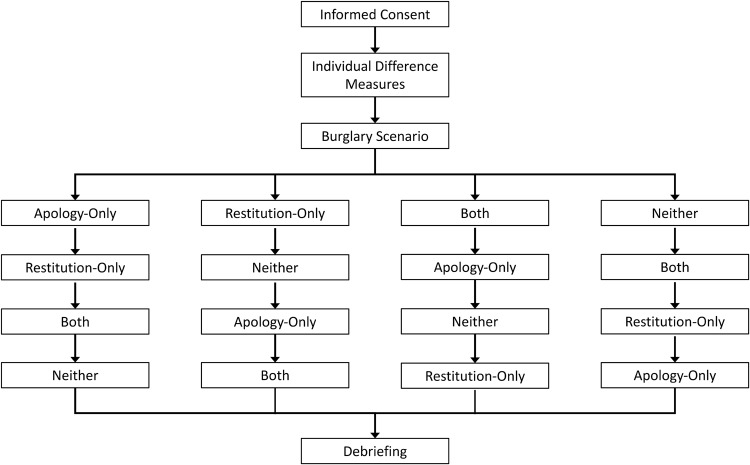
Study flow. Participants were randomly assigned to one of these four condition sequences. Participants first read and imagined each of the four condition scripts, completing the scales after each. Participants subsequently completed eight trials within each condition. Physiology was measured millisecond-to-millisecond and heartbeat-to-heartbeat throughout, and ratings were provided at the end of each condition.

As shown in Figure 1, participants were presented with a burglary scenario and four possible apology and restitution outcome scenarios (Apology-Only, Restitution-Only, Both, and Neither), which were adopted from Witvliet et al. (2020) and are described in detail below. In this within-subjects design, each participant imagined all conditions, with orders systematically counterbalanced, within males and within females. Participants were instructed to “try to vividly imagine these events as if they were actually happening to you right now” and to “focus on the thoughts, feelings, and physical reactions you would be having if this really happened to you.” This method has been used in both autobiographical (Witvliet et al., 2001) and scenario-based (Witvliet et al., 2008b) research paradigms.[Fn footnote1]

#### Stimulus Materials of Crime Description

In the burglary incident scenario, participants imagined that they had returned to their residence and discovered that it had been broken into. Personal items had been stolen, including $50 in cash, loose change, a watch, a treasured keepsake from a loved one, and their credit cards. Police investigated but did not apprehend anyone for the crime.

#### Stimulus Materials of Post-burglary Outcomes

Participants were randomly assigned to one of the Latin square condition sequences of the four possible outcomes: receiving an apology, restitution, both, or neither. The scenarios were designed to bear similarity in structure. Each script began: “You have not had the chance to replace all your stolen cards and IDs due to your busy schedule…”

##### Apology-only

“…The day after hearing from the detective, you receive a small, white envelope in the mail with no return address. Curious, you open the envelope. Inside is a folded piece of paper that you take out and open up. It is a note to you. The note says, ‘…I want to apologize to you. It was wrong of me to break into your apartment and take your things. Ever since, I have felt terrible. My conscience is really eating at me. I just wanted to tell you how bad I feel and how sorry I am that I probably have inconvenienced you to no end. If I had to do it over, I would ask for help instead of stealing. I wish I had never done it, and I know I never will do anything like this again. I am so sorry.”’

##### Restitution-only

“…The day after hearing from the detective, you receive a small, brown package in the mail with no return address. Curious, you open the package. Inside, you find your wallet, your watch, and a small envelope. You first check your wallet and find that the contents are all intact, including IDs, credit cards, pictures, and cash. Next you open the small envelope and find sixty dollars in cash and a note. The note says, ‘Here is your stuff and some money to make up for any trouble I caused you.”’

##### Both (apology and restitution)

The script for this condition began with the wording of the restitution condition, and then the wording of the note matched the apology condition.

##### Neither apology nor restitution

“…The day after hearing from the detective, you decide that you finally need to follow up with replacing your missing things. You travel to a local department store and purchase another watch. You go back to school and replace your ID card. Then, you drive back to the motor vehicle administration to get a new driver’s license. Finally, you call your credit card companies and ask them how long it will take for your new cards to be sent to you. Running these errands takes you the whole day.”

### Dependent Measures

Participants completed the scales (Table 1) in the first portion of the study, and they provided physiology measures followed by ratings (Table 2) in the second portion of the study (see the Figure 1 note).

**TABLE 1:** Means, standard deviations, and $\eta_{\text{p}}^{2}$ estimates of effect sizes (ES) for the scales assessing dependent variables.

	Neither	Apology-only	Restitution-only	Both	Apology		Restitution		A × R	
	*M (SD)*	*M (SD)*	*M (SD)*		*F (df)*	*ES*	*F (df)*	*ES*	*F (df)*	*ES*
Unforgiveness (12–60)	40.0^a^	33.1^b^	28.1^c^	24.5^d^	83.0***	0.58	104.5***	0.64	9.7**	0.14
	(8.8)	(10.0)	(9.9)	(9.5)	(1,60)		(1,60)		(1,60)	
Empathy (8–48)	12.7	20.2	23.8	30.5	116.1***	0.66	111.2***	0.65	0.67^n.s.^	0.01
	(5.2)	(9.0)	(10.1)	(11.2)	(1,60)		(1,60)		(1,60)	
Forgiveness (6–30)	10.7	14.0	16.7	19.7	78.1***	0.57	130.7***	0.69	0.22^n.s.^	0.00
	(4.4)	(4.9)	(5.6)	(5.9)	(1,60)		(1,60)		(1,60)	

Unforgiveness was measured with the Transgressions-Related Interpersonal Motivations Inventory, empathy was assessed with the Empathy Adjectives Scale, and forgiveness was measured with the Positive Responses to the Offender scale. ^*a,b,c,d*^Within each dependent variable for which the Apology × Restitution × Time interaction was significant, superscripts that differ indicate that the means significantly differ at p ≤ 0.008 (Bonferroni-corrected α). All 0.95 CIs around the mean differences did not cross zero where traditional significance testing indicated differences. **p < 0.01, ***p < 0.001, ^*n*.*s*^p > 0.42.

**TABLE 2:** Means, standard deviations, and $\eta_{\text{p}}^{2}$ estimates of effect sizes for the single-item ratings associated with dependent variables.

Single-item ratings (0–20 scale)	Neither	Apology-only	Restitution-only	Both	Apology		Restitution		A × R	
	*M (SD)*	*M (SD)*	*M (SD)*	*M (SD)*	*F (df)*	*ES*	*F (df)*	*ES*	*F (df)*	*ES*
**Dimensional ratings**										
Valence	3.62^a^	8.53^b^	15.22^c^	16.60^c^	62.29***	0.52	221.33***	0.80	21.66***	0.28
	(3.95)	(4.17)	(3.18)	(3.83)	(1,57)		(1,57)		(1,57)	
Arousal	14.34	11.57	10.05	9.03	11.88***	0.17	19.82***	0.26	3.29^n.s.^	0.06
	(5.62)	(5.17)	(5.70)	(6.32)	(1,57)		(1,57)		(1,57)	
Control	7.07	8.90	11.51	13.44	17.62***	0.23	53.74***	0.47	0.01^n.s.^	0.00
	(5.49)	(4.87)	(3.91)	(5.01)	(1,60)		(1,60)		(1,60)	
**Negative emotions**										
Anger	16.20^a^	12.78^b^	7.23^c^	5.40^c^	25.82***	0.30	136.64***	0.70	3.92^∗^	0.06
	(4.09)	(5.26)	(5.03)	(5.03)	(1,59)		(1,59)		(1,59)	
Sadness	11.13	9.47	5.30	4.22	8.64**	0.13	61.52***	0.51	0.28^n.s.^	0.01
	(5.62)	(4.82)	(4.59)	(4.01)	(1,59)		(1,59)		(1,59)	
Fear	8.05	5.76	4.83	3.17	22.57***	0.28	31.68***	0.35	0.57^n.s.^	0.01
	(5.32)	(4.63)	(4.53)	(3.17)	(1,58)		(1,58)		(1,58)	
**Positive emotions**										
Gratitude	3.00^*a*^	6.97^ b^	15.10^*c*^	16.40^*d*^	30.78***	0.34	264.71***	0.82	10.63**	0.15
	(3.99)	(5.58)	(3.82)	(4.42)	(1,59)		(1,59)		(1,59)	
Empathy	3.52	7.80	9.43	12.54	59.71***	0.50	64.73***	0.52	2.23^n.s.^	0.04
	(3.75)	(4.96)	(5.29)	(5.10)	(1,60)		(1,60)		(1,60)	
Forgiveness	5.28	9.08	12.58	15.10	47.13***	0.44	110.78***	0.65	2.79^n.s.^	0.05
	(5.00)	(5.65)	(4.78)	(4.64)	(1,59)		(1,59)		(1,59)	

^*a,b,c,d*^Within each dependent variable that has a significant Apology × Restitution interaction, superscripts that differ indicate that the means significantly differ at p ≤ 0.008 (Bonferroni-corrected α). Where differences are noted, the 0.95 CIs around mean differences also did not cross zero. The comparisons of ratings values for Restitution-Only and Both were marginally different for both anger and valence (both ps = 0.01). ^∗^p < 0.05, **p < 0.01, ***p < 0.004.

#### Self-Report Measures

Participants completed the Transgression-Related Interpersonal Motivations Inventory (TRIM; McCullough et al., 1998) as a measure of unforgiveness, the Empathy Adjectives Scale as a measure of empathy (Batson et al., 1986), and Positive Responses to an Offender (PRO), which is appropriate for non-close relationships (Witvliet et al., 2008b, 2020), as a measure of forgiveness. Participants rated their level of emotional valence (negative–positive), arousal (low–high), and perceived control by using a joystick to manipulate an androgynous line drawing of a person to display the affect they felt (see Hodes et al., 1985). Then, they used the joystick to register along a continuous line (labeled from not at all to moderately to completely) their own levels of fear, sadness, anger, gratitude, empathy for the perpetrator, and forgiveness for the perpetrator. All ratings were converted to a scale ranging from 0 to 20. The order of questions was systematically varied within and across participants.

#### Physiology

We continuously measured participants’ second-by-second facial EMG activity above the brow at the CS muscle and under the eye at the OO muscle. On a beat-to-beat basis, we measured HR and systolic blood pressure, multiplying them to derive rate pressure products, which are indicative of cardiac stress (see Kitamura et al., 1972; Lawler et al., 2003; Lawler-Row et al., 2008; Witvliet et al., in press).

### Apparatus

Participants sat in a recliner in a private room. To time the presentation of tones and collect on-line physiological data, we used a Dell 486 computer and VPM software (Cook et al., 1987). To measure participant ratings after imagery, we used a joystick and a second Dell 486 with VPM software in the participant room. Imagery and relaxation trials were signaled by auditory tones at two frequencies—high (1,350 Hz) and low (620 Hz), respectively. The tones were 500 ms long and 73 dB[A]. Before the physiological data collection portion of the experiment, participants heard the tones to ensure they could distinguish between them.

Facial EMG was recorded at the CS (i.e., brow) and OO (i.e., under the eye) muscle regions using sensor placements suggested by Fridlund and Cacioppo (1986). Miniature Ag-AgCl electrodes filled with electrode gel were applied. EMG signals were amplified (50,000×) by a Hi Gain V75-01 bioamplifier, using 90 Hz high-pass and 1 kHz low-pass filters. Signals were rectified and integrated by a Coulbourn multifunction V76-23 integrator (nominal time constant = 10 ms).

For HR, electrocardiogram data were collected using two standard electrodes, one on each forearm. A Hi Gain V75-01 bioamplifier amplified and filtered the signals. The signals were then sent to a digital input on the computer that detected R waves and measured interbeat intervals in milliseconds. Data were converted off-line to beats per minute. To derive rate pressure product data, we used the HR data and the systolic blood pressure data measured by a Colin 7000 continuous non-invasive blood pressure monitor with a solid-state pressure transducer array attached to the wrist. This provided continuous, beat-to-beat blood pressure values. An oscillometric cuff was used to provide calibration for the wrist transducer array during a 10 min initial calibration period preceding the physiology section of each experimental session.

### Procedure

Participants attended a 2 h testing session (Figure 1). In the first portion of the study, participants provided individual difference information including demographic data and scales to assess religious commitment, forgivingness, anger, and rumination (see Witvliet et al., 2008a). State scales measuring unforgiveness, empathy, and forgiveness followed the burglary incident scenario and the possible outcomes. To control for effects related to the order of conditions, we used a Latin square design, with participants randomly assigned to one of four orders:

(a) Apology-Only → Restitution-Only → Both → Neither
(b) Restitution-Only → Neither → Apology-Only → Both
(c) Both → Apology-Only → Neither → Restitution-Only
(d) Neither → Both → Restitution-Only → Apology-Only

For the psychophysiology measurement in the second portion of the study, the participants retained the same Latin square sequence of conditions (Apology-Only, Restitution-Only, Both, or Neither). Within each condition, participants completed a block of eight trials; this helped them focus and reduced potential interference from the other conditions.

**FIGURE 2:**
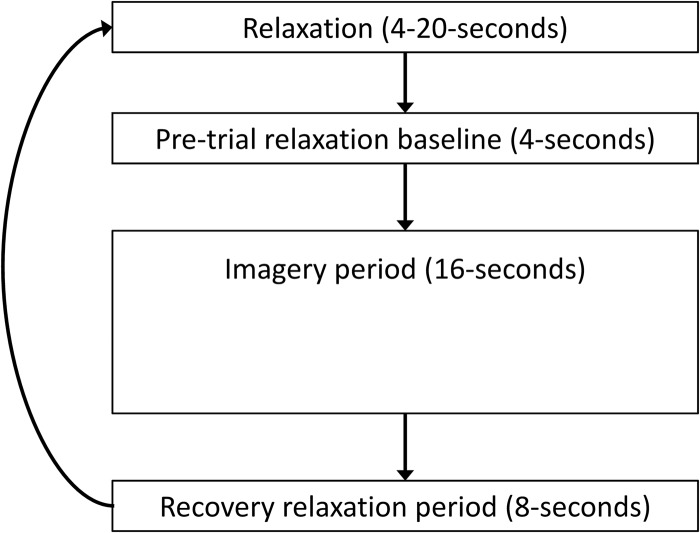
Schematic for a trial of relaxation, pretrial baseline, imagery, and recovery. The participant began with several relaxation periods and then completed eight of these trials within each of the four conditions (Apology, Restitution, Both, and Neither). After the final trial for that condition, the participant rated the emotions experienced during imagery for that condition.

Thus, within each condition (e.g., Apology-Only), the participant completed eight trials. A sample trial is shown in Figure 2. A low-pitched tone signaled the participant to relax by thinking the word one every time he or she exhaled (e.g., Witvliet and Vrana, 1995). The relaxation period before imagery allowed us to measure that trial’s baseline physiology data. Then, a high-pitched tone signaled the participant to imagine the scenario for that condition (e.g., Apology-Only). The imagery period was followed by a relaxation period that allowed us to measure physiological recovery. A variable number of relaxation periods occurred, such that a range from 16 to 32 s of relaxation occurred between imagery periods, to ensure that participants relaxed and to reduce the predictability of what was coming next. By the end of the physiology portion of the study, participants had imagined each condition scenario eight times, for a total of 32 imagery trials. As Figure 2 shows, physiological responses were measured continuously during 4 s baseline (relaxation), 16 s imagery, and 8 s recovery periods. Following each block of eight imagery trials within a condition, participants used a video display and computer joystick to rate their emotional responses privately and record them directly into a computer.

### Data Reduction

**FIGURE 3:**
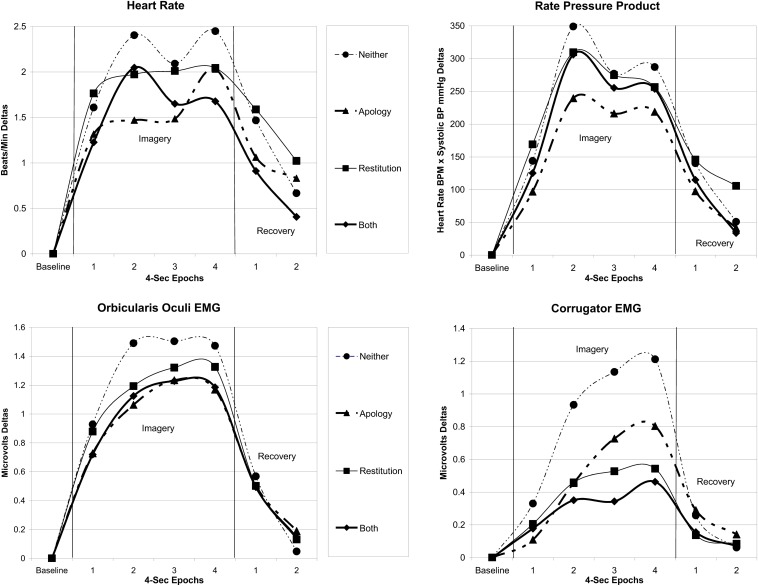
Physiological reactivity and recovery patterns in the sample are depicted to show difference scores for imagery and recovery periods (i.e., with the 4 s pretrial baseline value for that imagery and recovery trial subtracted) by condition over time. The 4 s baseline value is set to zero, so that changes through four 4 s epochs during active imagery and two 4 s epochs of relaxation during the recovery period are clearly shown. Within each of the four conditions, the data were averaged across eight trials.

We used a standard approach to reduce the physiology data and compute a difference-from-baseline metric for each trial within each participant (see Witvliet and Vrana, 1995; Witvliet et al., 2001, 2008b). For each physiology measure (CS, OO, HR, and rate pressure product), each of the 32 trials had its own baseline (4 s), imagery (16 s), and recovery period (8 s). Data were averaged in 4 s epochs (one for baseline, four for imagery, and two for recovery). For each of the epochs during imagery and recovery, deltas were created by subtracting from each epoch that particular trial’s baseline data. This approach offers correction to the reference value from the pretrial baseline level, highlights the directional effects of the conditions on each physiological measure (e.g., increases or decreases), and reduces variance due to movement and habituation that can make raw scores particularly difficult to interpret meaningfully. The deltas across the epochs of the eight trials within each of the conditions (Apology-Only, Restitution-Only, Both, and Neither) were averaged and are plotted in the panels of Figure 3.

### Statistical Analyses

For each Apology × Restitution condition, all imagery and recovery epoch deltas, as described in the above data reduction section, were averaged. We then used SPSS to run 2 Apology (present and absent) × 2 Restitution (present and absent) repeated-measures ANOVAs for imagery data and recovery data. We interpreted the results using the multivariate tests because they do not assume sphericity (see Green et al., 2000, p. 213). The F-statistic equivalent for Wilks’s lambda is reported for each physiology measure during imagery and for each self-report rating. Whenever an Apology × Restitution interaction was found, all combinations of the four conditions (Apology-Only, Restitution-Only, Both, and Neither) were compared using six paired-samples *t*-tests with *p*-values corrected using the Bonferroni procedure (critical *p* = 0.05/6 = 0.008); to evaluate simple effects in the presence of a statistically significant interaction effect, 0.95 confidence intervals around mean differences were also examined.

## Results

We report means, standard deviations, and effect sizes for the 2 Apology × 2 Restitution repeated-measures analyses of variance for state self-report scales in Table 1 and ratings in Table 2. Physiological patterns during imagery and recovery are depicted in Figure 3. We verified that participants randomly assigned to each counterbalanced condition order did not differ in their unforgiving, empathic, and positive responses to the offender after reading the crime incident (all scale score *F*s ≤ 1.11, *p*s ≥_0.355). As a manipulation check, participants provided ratings, with all participants indicating that they could imagine the crime scenario actually happening to them at least a little, 38% reporting that the scenario reminded them of a situation in their lives, and 21% reporting having personally experienced the crime in real life. Those who had and had not experienced a burglary were statistically equivalent across dependent variables; we therefore retained all participants.

Consistent with predictions, apology and restitution each had main effects of decreased unforgiveness (i.e., TRIM scores) and increased empathy and positive responses toward the offender (i.e., forgiveness as assessed on the PRO; see Table 1). An Apology × Restitution interaction was significant only for unforgiveness scores on the TRIM. Both apology-only and restitution-only reduced unforgiveness compared to neither; additionally, the combination of both apology and restitution reduced unforgiveness to a greater degree than either one alone. However, the impact of restitution was greater than the impact of apology for reducing unforgiveness.

All single-item emotion ratings (see Table 2) showed the predicted main effects of apology and restitution. Participants reported significantly lower levels of anger, fear, and sadness; significantly higher levels of gratitude, empathy, and forgiveness toward the perpetrator; and significantly greater perceived control when either an apology or restitution was present versus absent. Participants also rated their emotions during imagery as more positively valent and less aroused (more calm) when an apology or restitution was present compared to when each was not.

Three ratings showed the interactive effects of apology and restitution. *Post hoc* analyses showed that apology-only and restitution-only reduced anger while also elevating positive valence and gratitude compared to neither an apology nor restitution. However, different patterns emerged for the condition in which both an apology and restitution were received. For anger and valence, the presence of both an apology and restitution combined did not yield different results from the restitution-only condition. For gratitude, by contrast, the effects of apology and restitution were additive, with the combination of both apology and restitution prompting greater gratitude than apology-only or restitution-only.

### Imagery Session Physiology^2^ (See Figure 3)

#### Main Effects of Apology and Restitution

During imagery, the conditions in which an apology was present versus absent were associated with less reactivity (smaller deltas) for HR, *F*(1,60) = 5.52, *p* < 0.022, $\eta_{\text{p}}^{2}$ = 0.08; rate pressure products, *F*(1,54) = 5.39, *p* = 0.024, $\eta_{\text{p}}^{2}$ = 0.09; and OO muscle activity under the eye, *F*(1,59) = 6.31, *p* = 0.015, $\eta_{\text{p}}^{2}$ = 0.10. Restitution did not have a statistically significant main effect on any of these measures, *F*s < 0.640, *p*s > 0.43. Rate pressure product levels also continued to be significantly less elevated in the recovery period following conditions in which an apology was received, *F*(1,54) = 21.06, *p* < 0.001, $\eta_{\text{p}}^{2}$ = 0.28. For all other dependent measures, no statistically significant differences occurred during the recovery period, *F*s < 2.40, *p*s < 0.13.

Corrugator supercilii (CS) muscle activity at the brow was significantly lower during imagery of receiving (versus not receiving) either an apology, *F*(1,58) = 4.83, *p* = 0.032, $\eta_{\text{p}}^{2}$ = 0.08, or restitution, *F*(1,58) = 17.65, *p* < 0.001, $\eta_{\text{p}}^{2}$ = 0.23. Lower CS activity after restitution continued as a trend in the recovery period, *F*(1,58) = 3.00, *p* = 0.089, $\eta_{\text{p}}^{2}$ = 0.05.

#### Interactive Effects of Apology and Restitution

Only one physiological measure, CS brow muscle activity during imagery, was influenced by an Apology × Restitution interaction, *F*(1,58) = 4.00, *p* = 0.05, $\eta_{\text{p}}^{2}$ = 0.07. The presence of restitution-only was more potent than apology-only for quelling activity at the brow muscle during imagery (see Figure 3, corrugator panel). Similar to valence and anger, the presence of restitution was so potent for CS activity that the addition of an apology did not yield further change.

## Discussion

This experiment provides the first psychophysiological investigation of the presence of apology and of restitution as conditions that predict reduced unforgiveness and elevated forgiveness responses. This experiment marshaled self-report and physiological evidence for the roles that a thorough apology and restitution can play in promoting affective change consistent with emotional forgiveness (Worthington and Scherer, 2004; Worthington, 2006; Witvliet et al., 2010). Because a major contribution to the literature is an analysis of the physiological findings, we discuss this first.

### Psychophysiological Changes

Apology and restitution—which signal accountability through a perpetrator’s relational and reparative responses after wrongdoing—each subdued activity at the CS brow muscle and also decreased the negativity of valence ratings, as predicted (see Witvliet and Vrana, 1995). Furthermore, a significant interaction showed that restitution was so potent in decreasing CS activity—while elevating positive valence and diminishing anger—that the further addition of an apology did not yield additional change.

Only the presence of an apology calmed activity at the OO muscle under the eye as well as HR reactivity. Past research manipulating the affective arousal and valence of imagery found that emotionally high (vs. low) arousal conditions prompted greater OO activity regardless of valence (Witvliet and Vrana, 1995). In addition to its calming effect during imagery, an apology was associated with less cardiovascular stress and myocardial oxygen demand in the recovery periods, as indicated by the rate pressure product level deltas (see Kitamura et al., 1972).

These data complement results from laboratory investigations that have associated apologies with improved cardiovascular recovery (Anderson et al., 2006; Whited et al., 2010; Kubo et al., 2012). Importantly, Schwartz et al. (2003) have assembled a strong theoretical case that cardiovascular reactivity may play a role in long-term health effects. Persistent activation in the absence of stressors (e.g., elevated rate pressure products, impaired HR variability during the recovery period) is more likely to accumulate in adverse effects over time. Prior research has shown that rumination about an interpersonal offense reliably impairs HR variability, an indicator of vagal tone and parasympathetic activity (Witvliet et al., 2010, 2011). Unforgiveness—when chronic—has been hypothesized to increase the risk of coronary heart disease (for a review, see Harris and Thoresen, 2005) and stress-related disorders (for a review, see Witvliet et al., in press).

### Self-Reported Evidence for Emotional Change

Apology and restitution had independent and interactive effects on self-reports related to forgiveness and emotional change, which replicate and extend self-report research (Witvliet et al., 2020). Both apology and restitution significantly decreased the negativity of valence ratings and the intensity of arousal ratings. These collective changes in response to the hypothetical scenario are consistent with the emotional differences induced by focusing on unforgiving versus forgiving imagery about a real-life offender (Witvliet et al., 2001).

Apology and restitution also reliably reduced unforgiveness (i.e., TRIM scores) and ratings of anger, sadness, and fear. Furthermore, apology and restitution increased scores across gratitude, empathy, and forgiveness measures. These patterns are consistent with Worthington and Wade’s (1999) hypothesis that emotional forgiveness involves supplanting negative unforgiving emotions with positive other-oriented emotions. The conditions that promoted forgiveness also increased perceived control, paralleling findings for state forgiveness in response to both real-life transgressors (Witvliet et al., 2001) and a scenario-based criminal offender (Witvliet et al., 2008b).

Interactions of apology and restitution pointed to the potency of restitution-only beyond apology-only to reduce unforgiveness and anger, while elevating gratitude and positive valence. In particular, valence and anger were so responsive to restitution that the addition of an apology did not further elevate the positivity of participants’ emotion or decrease their anger ratings. Similarly, the CS brow muscle was so responsive to the presence of restitution that adding an apology did not further subdue activity there.

This study of perpetrator response effects also emphasizes gratitude. The even stronger impact of restitution-only than apology-only on gratitude is consistent with Emmons and Shelton’s (2002) view that gratitude intensifies when a personal, positive outcome—such as tangible restitution—clearly results from the actions of another. This pattern echoes findings by Emmons and McCullough (2003) that when people focus on benefits even in adversity (e.g., restitution after an injustice in our study), they experience greater gratitude and a range of emotional benefits. Furthermore, gratitude has been shown to reduce negative affect (McCullough et al., 2004), and benefit-focused reappraisal after an offense has been found to generate gratitude while fostering forgiveness along with emotional and cardiovascular regulation (Witvliet et al., 2010). This experiment points to gratitude as an important topic to advance research on justice, accountability, and forgiveness.

The current experiment provides affective and stress-related physiological responses that replicate and extend self-report responses (Witvliet et al., 2020) and that align with coded behavioral (and self-report) responses in a role-play simulation (Kiefer et al., 2020). Apologies and restitution represent verbal relational and tangible recompense indications of offenders’ accountable responsibility-taking for an injustice toward a victim, and they have the capacity to evoke increases in forgiveness with emotional and embodied change.

### Limitations and Suggestions for Future Research

The use of hypothetical crime offenses rather than an autobiographical one allowed us to increase internal validity by exerting control over the content of the offense, ensure that the offense was blameworthy and one-sided with quantifiable restitution, and extend the literature programmatically (Witvliet et al., 2008b, 2020). A limitation of hypothetical offenses is that they can lack ecological validity and psychological realism or reduce participant involvement. However, all of the present participants reported being able to imagine the scenario actually happening to them, with nearly two in five participants indicating that it was similar to a situation they experienced and one in five reporting past experience with being burglarized. Statistical tests showed that participants who did and did not personally experience the crime gave similar responses.

Kiefer et al. (2020) have begun to expand on the ecological validity in apology and restitution research by studying role-play simulations of restorative justice using family group conferencing. Groups of four people were comprised of an offender (male), a victim (male), the offender’s mother, and the victim’s mother. Each quartet saw a video of lawyers tell about the crime and its effects from both sides. Before engaging in 30 min of mediated role-play, the offender was randomly assigned to apologize with an offer of restitution or forbidden from doing so. Responses of each of the three other participants in each quartet were analyzed. Generally, the victim and the victim’s mother forgave more in the apology-with-restitution condition than in the no-apology and no-restitution condition. However, the offender’s mother was equally likely to forgive her son in each condition—and less likely to forgive than either the victim or the victim’s mother. Although role-play simulations have their own challenges in ecological validity, this role-play simulation’s results aligned with and extend the present findings. A valuable next step would be to study apology and restitution in the context of actual restorative justice mediations (Armour and Umbreit, 2006). Such work could incorporate ambulatory physiology monitoring and daily diary methods to assess psychophysiology.

In light of restorative justice research, one current finding especially warrants follow-up research. Restitution was associated with reductions in negative affective self-reports and facial expressions at the corrugator. Why was restitution—which also reduced arousal—not associated with changes in OO activity under the eye (Witvliet and Vrana, 1995) or significant cardiovascular effects? One clue may be found in the justice results found by Witvliet et al. (2008b). In that study, signs of cardiovascular stress (rate pressure products) were lower for retributive versus no justice, but not for restorative justice, which included perpetrator remorse and restitution. The current experiment parsed apology and restitution, linking cardiovascular stress reduction to apology, whereas the apology was not strongly manipulated by Witvliet et al. (2008b). Future work could investigate why the apology condition reliably calmed cardiovascular stress and arousal under the eye, whereas restitution responses did not. One possibility is that in this context, the apology signaled perpetrator empathy and social support for the victim, which may have reduced stress-related physiology even more than tangible recompense. Understanding this will have implications for emerging understandings of justice and its relationship to victim well-being and health.

## Conclusion

This work provides psychophysiological evidence for the effectiveness of apology and restitution in facilitating empathy and forgiveness in a context that did not excuse injustice (Worthington, 2006). Findings are consistent with theorizing about accountability (Witvliet, 2020) and the injustice gap generated by transgressions (Exline et al., 2003). This work also advances the relatively under-examined domain of antecedents to forgiveness that have implications for psychophysiological side effects. In turn, these side effects can serve as pathways to health. Apology and restitution are important elements indicative of perpetrator accountability and are relevant to restorative justice (Petrucci, 2002; Witvliet et al., 2008b; Kiefer et al., 2020). Thus, it may be fruitful to identify ways in which the willingness to engage in accountable repair responses toward victims of wrongdoing may be linked to empathic and self-regulatory mechanisms that may undergird both repentant change in perpetrators and responsible forgiveness in victims.

## Data Availability Statement

The studies involving human participants were reviewed and approved by the Human Subjects Review Board, Hope College. The participants provided their written informed consent to participate in this study.

## Ethics Statement

All participants provided written informed consent to the study procedure, which was approved by the local Ethics Committee (Hope College).

## Author Contributions

CW and EW conceived the study. CW programmed the study, collected and analyzed data, and drafted the manuscript. LR was involved in the data collection and cross-checking of analyses. CW, LR, EW, and J-AT contributed to conceptualizations of the findings, substantively revised the manuscript, and read and approved the submitted version.

## Conflict of Interest

The authors declare that the research was conducted in the absence of any commercial or financial relationships that could be construed as a potential conflict of interest.

## Footnotes

1 A separate project analyzed religious commitment correlations in a subset of the sample who self-identified as Christian, focusing on traits of forgivingness, empathy, rumination, and anger, as well as states associated with the crime incident and control condition that lacked a perpetrator repair response (Witvliet et al., 2008a).

2 No other main effects were found for the physiological measures (all Apology *F*s ≤ 2.40, all *p*s ≥ 0.13; all Restitution *F*s ≤1.6, all *p*s ≥ 0.20), and no other interactions during imagery or recovery periods were statistically significant for other physiology measures (all Apology × Restitution *F*s ≤ 2.43, all *p*s ≥ 0.13), including other measures of zygomatic EMG, skin conductance levels, and blood pressure. Due to space constraints, n.s. reports are not in the text. This study was designed to balance the number of male and female participants, although not intended to test sex effects. Because Whited et al. (2010) found that participant sex interacted with a between-subjects lab-based apology for diastolic and mean arterial blood pressure, we conducted similar *post hoc* analyses. We tested the absence vs. presence of apology within participants using the Neither and Apology-Only conditions, with sex as the between-participant grouping variable. A significant Apology × Sex interaction, *F*(1,53) = 5.45, *p* = 0.023, $\eta_{\text{p}}^{2}$ = 0.09, occurred only for mean arterial pressure imagery deltas during imagery. The pattern of means aligned with the finding of Whited et al. (2010), such that that mean arterial pressure was higher in the males in the apology present condition, whereas females showed the opposite pattern. In the current study, males’ mean arterial pressure deltas were reliably higher during apology-only imagery than no-apology imagery, but females’ decrease in mean arterial pressure reactivity in response to the presence of an apology was not reliable.
